# Comparative functional pan-genome analyses to build connections between genomic dynamics and phenotypic evolution in polycyclic aromatic hydrocarbon metabolism in the genus *Mycobacterium*

**DOI:** 10.1186/s12862-015-0302-8

**Published:** 2015-02-14

**Authors:** Ohgew Kweon, Seong-Jae Kim, Jochen Blom, Sung-Kwan Kim, Bong-Soo Kim, Dong-Heon Baek, Su Inn Park, John B Sutherland, Carl E Cerniglia

**Affiliations:** Division of Microbiology, National Center for Toxicological Research/FDA, Jefferson, Arkansas USA; Center for Biotechnology, Bielefeld University, Bielefeld, Nordrhein-Westfalen Germany; Department of Management, University of Arkansas at Little Rock, Little Rock, Arkansas USA; Department of Life Science, Hallym University, Chuncheon, Gangwon-do 200-702 Republic of Korea; Department of Oral Microbiology and Immunology, School of Dentistry, Dankook University, Chonan, Republic of Korea; Department of Computer Science and Engineering, Texas A&M University, College Station, Texas USA

**Keywords:** *Mycobacterium*, PAH metabolism, Pan-genome, Functional genomics, Functional pan-genome, Phenotype network, Evolution, Epistasis, Pleiotropy

## Abstract

**Background:**

The bacterial genus *Mycobacterium* is of great interest in the medical and biotechnological fields. Despite a flood of genome sequencing and functional genomics data, significant gaps in knowledge between genome and phenome seriously hinder efforts toward the treatment of mycobacterial diseases and practical biotechnological applications. In this study, we propose the use of systematic, comparative functional pan-genomic analysis to build connections between genomic dynamics and phenotypic evolution in polycyclic aromatic hydrocarbon (PAH) metabolism in the genus *Mycobacterium*.

**Results:**

Phylogenetic, phenotypic, and genomic information for 27 completely genome-sequenced mycobacteria was systematically integrated to reconstruct a mycobacterial phenotype network (MPN) with a pan-genomic concept at a network level. In the MPN, mycobacterial phenotypes show typical scale-free relationships. PAH degradation is an isolated phenotype with the lowest connection degree, consistent with phylogenetic and environmental isolation of PAH degraders. A series of functional pan-genomic analyses provide conserved and unique types of genomic evidence for strong epistatic and pleiotropic impacts on evolutionary trajectories of the PAH-degrading phenotype. Under strong natural selection, the detailed gene gain/loss patterns from horizontal gene transfer (HGT)/deletion events hypothesize a plausible evolutionary path, an epistasis-based birth and pleiotropy-dependent death, for PAH metabolism in the genus *Mycobacterium*. This study generated a practical mycobacterial compendium of phenotypic and genomic changes, focusing on the PAH-degrading phenotype, with a pan-genomic perspective of the evolutionary events and the environmental challenges.

**Conclusions:**

Our findings suggest that when selection acts on PAH metabolism, only a small fraction of possible trajectories is likely to be observed, owing mainly to a combination of the ambiguous phenotypic effects of PAHs and the corresponding pleiotropy- and epistasis-dependent evolutionary adaptation. Evolutionary constraints on the selection of trajectories, like those seen in PAH-degrading phenotypes, are likely to apply to the evolution of other phenotypes in the genus *Mycobacterium*.

**Electronic supplementary material:**

The online version of this article (doi:10.1186/s12862-015-0302-8) contains supplementary material, which is available to authorized users.

## Background

*Mycobacterium* is the single genus in the family *Mycobacteriaceae*, in the order *Actinomycetales* [1]. There are more than 150 recognized parasitic and free-living species of *Mycobacterium* (http://www.bacterio.cict.fr/m/mycobacterium.html) with remarkable metabolic and physiologic versatility, enabling colonization of diverse habitats [2,3]. There has been great interest in mycobacteria due to their abilities both to cause devastating diseases in humans and animals and to degrade toxic compounds in the environment [4,5]. For these reasons, the number of available genome sequences has been growing fast and genome-scale omics data have been generated, allowing deeper functional genomics insights into their phenotypic features [6-13]. Several mycobacterial models have played an important role in the functional genomics understanding of basic phenotypic traits. For example, *M. tuberculosis* and *M. leprae*, for mycobacterial pathogenicity, and *M. vanbaalenii* PYR-1, for high-molecular-weight (HMW) PAH biodegradation, have been studied and the accumulated information has been applied to obtain information on other species [3,5,14,15]. However, despite the exciting discoveries continually adding to the broader and deeper knowledge of mycobacteria, there is a significant knowledge gap between genome and phenome in the genus *Mycobacterium*, due mainly to the epistatic and pleiotropic complexity of the genomic and phenotypic traits. The epistatic interaction—functional combination of two or more genes (or enzymes) for a single trait (or phenotype)—and pleiotropic activity—functional contribution of a gene (or enzyme) for multiple traits (or phenotypes)—are still poorly understood at the genome level [16] but are essential for a deeper understanding of mycobacterial systems.

**Figure 1:**
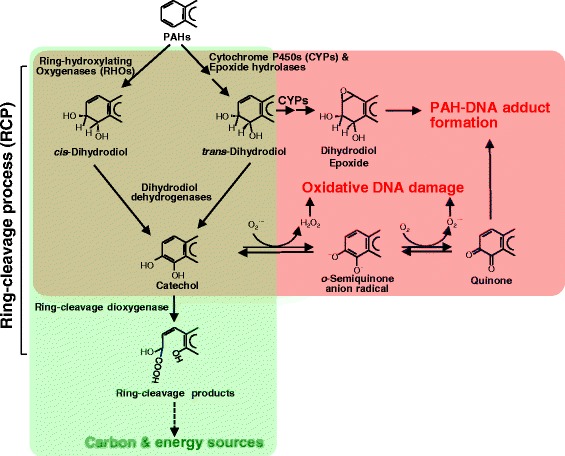
**Metabolic ambiguity of PAHs as nutrient or toxicant**. The ring-cleavage process (RCP)—mono- or dioxygenation by ring-hydroxylating oxygenases (RHOs) or cytochrome P450 monooxygenases (CYPs), dehydrogenation by dihydrodiol dehydrogenase, and ring-cleavage by ring-cleavage dioxygenase—in mycobacterial PAH metabolism responsible for metabolic benefits is in the green colored area, while production of toxic PAH metabolites/byproducts and reactive oxygen species (ROS), which have the potential to cause PAH-adducts and oxidative DNA damage, is in the red colored area.

Newly arising genomic variations create phenotypic changes upon which natural selection can act. The evolutionary genetics of epistasis and pleiotropy not only make their effects on evolution difficult to predict, but also constrain the path of natural selection, which allows observation of only a small fraction of possible evolutionary trajectories [16]. In this respect, mycobacterial HMW PAH degradation is of particular interest, because of the metabolic ambiguity (as nutrient or toxicant) (Figure 1) and the environment-dependent availability of HMW PAHs [8]. As shown in Figure 1, PAHs serve not only as nutrients to be metabolized but also as potential toxicants if they are transformed to diol-epoxides by metabolic activation [17-19]. PAHs consist of two or more fused aromatic rings arranged in various configurations [20]. Although PAHs are introduced into the environment mainly from anthropogenic routes, they are also found occurring naturally in the environment. Since physical and chemical characteristics of PAHs vary with molecular weight, PAHs can be divided into two groups, low-molecular-weight PAHs (LMW PAHs), with two or three fused aromatic rings, and HMW PAHs, with four or more fused aromatic rings. HMW PAHs are more toxic and difficult to degrade, persisting longer than LMW PAHs in the environment [20]. Microbial metabolic activity represents one of the primary processes of degradation and transformation of PAHs in the environment [20], and some members of the genus *Mycobacterium* have been shown to be able to degrade HMW PAHs [12,21,22]. Therefore, a possible full evolutionary scenario for PAH metabolism in the genus *Mycobacterium* could provide deeper insights into the relationship between genomic variations and natural selection.

*M. vanbaalenii* PYR-1 has been extensively studied as a model microorganism for bacterial HMW PAH degradation at both laboratory and field scales [5,14,15]. Recently, a PAH metabolic network (PAH-MN), including HMW PAHs, has been systematically reconstructed using genomic, proteomic, and metabolic information [9]. According to the PAH-MN, PAH substrates are degraded by a set of interconnected functional processes, termed ring-cleavage processes (RCPs), side-chain processes (SCPs), and central aromatic processes (CAPs). Under the common aromatic metabolic logic (the activation of benzene rings and ring opening), the oxygen-dependent activation of thermodynamically stable benzene rings and ring cleavage occurs in RCP, the side chain processing of the opened ring to produce biological metabolic precursors occurs in SCP, and the metabolic connection of protocatechuate to the tricarboxylic acid (TCA) cycle occurs in CAP. In our previous study with *M. vanbaalenii* PYR-1, a fundamental building principle of the PAH-MN, a patchwork assembly of the phylogenetic modules, with a backward evolutionary direction from CAP via SCP to RCP, has been hypothesized on the basis of the functional association of the phylogenetic modules [9]. When considering the ambiguous metabolic effects of HMW PAHs, the model microorganism can benefit from the buildup of the PAH-MN in both architectural and functional aspects. That is, the directed growth of the metabolic network from CAP via SCP to RCP increases the architectural and functional connectivity among phylogenetic modules with PAH-degrading enzymes, resulting in decreasing the epimetabolome, including toxic intermediates, such as diol epoxides [9]. Therefore, the backward evolutionary growth of the PAH-MN can also have a practical toxicological benefit that minimizes toxic effects of the activated PAH intermediates, which are produced in RCP. As revealed in the proteomic analysis of the PAH-MN, function-specific regulation of the functional modules (RCP, SCP, and CAP) completes the evolutionary and metabolic endeavors for nutritional benefits from PAHs, by allowing mature metabolic behavior of the channel management for global PAH metabolism with more productivity and less toxicity (that is, generating more productive biological precursors, such as pyruvate and acetyl-CoA, and fewer toxic intermediates, such as *o*-quinones) [9]. Therefore, it is evident that mycobacterial PAH metabolism is an epistasis-centric phenotype, which is essential for functional combination of two or more genetic modules and requires active investment to obtain and continuous, sophisticated management to keep. However, no pan-genomic evidence has been evaluated for this hypothesis, and consequently, a full evolutionary story of the PAH-degrading phenotype in the genus *Mycobacterium* has not been proposed.

Phenotypic traits of living cells are the result of successful functional interactions among biochemical compounds in highly complex biochemical networks [9,23]. It is a major challenge to understand causes and consequences of evolution of the phenotypes. In this study, to retrace plausible evolutionary trajectories of PAH metabolism in the genus *Mycobacterium* with no anthropomorphic bias, we employed three key concepts: networks, pan-genomic comparison, and functional genomics. First, networks allow for intuitive platforms for connection and visualization of the interacting parts with a more holistic view of phenotypic interactions in the MPN and functional interactions of the genetic modules in the PAH-MN. Second, pan-genomic comparison of the mycobacterial genomes, which exhibit extreme levels of evolutionary plasticity with high levels of gene gain and loss during evolution [24], allows integrating the genome sources to increase the power of genomic analyses. Finally, functional genomic data (proteomic data in this study) filter out the static genes that are not expressed and so may not be involved in a given phenotype from the pan-genomic data to provide phenotype-related functional pan-genomic dynamics. This multidisciplinary approach, which requires intensive systematic integration of the three different types of data, was essential to elucidate the nature and underlying mechanisms, and the phenotypic feedbacks, of epistatic and pleiotropic evolutionary effects of PAH metabolism in the genus *Mycobacterium*.

## Results and discussion

### Systematic integration of data

In this study, initially, phylogeny, phenotypes, and genomic contents of 27 completely genome-sequenced mycobacteria were systematically integrated to generate an applicable mycobacterial compendium of phenotypic and genomic properties (Table 1). On the basis of the integrated information, an MPN was reconstructed and analyzed from a pan-genomic perspective. A series of consecutive functional pan-genomic analyses provided conserved and unique genomic evidence for strong epistatic and pleiotropic impacts on the evolutionary trajectories of the PAH-degrading phenotypes in the genus *Mycobacterium*.

**Table 1:** **Phenotypic and genomic properties of the 27 completely genome-sequenced strains of mycobacteria**

**Mycobacterial strain**	**Phenotypic properties**				**Genomic properties**				**Reference**
	**Life-style** ^***a***^	**Growth rate**	**Pathogenicity**	**PAH-metabolism**	**Genome size**	**GC %**	**Gene count**	**COG count**	
*Mycobacterium smegmatis* MC^2^ 155	FL	Fast	Non-pathogenic	PAH-non-degrading	6988209	0.67	6941	4796	[25]
*Mycobacterium vanbaalenii* PYR-1	FL	Fast	Non-pathogenic	PAH-degrading	6491865	0.68	6047	4186	[26,27]
*Mycobacterium* sp. KMS	FL	Fast	Non-pathogenic	PAH-degrading	6256079	0.68	6089	4135	[12]
*Mycobacterium* sp. JLS	FL	Fast	Non-pathogenic	PAH-degrading	6048425	0.68	5855	4148	[12]
*Mycobacterium* *gilvum* PYR-GCK	FL	Fast	Non-pathogenic	PAH-degrading	5982829	0.68	5683	3901	[28]
*Mycobacterium* sp. MCS	FL	Fast	Non-pathogenic	PAH-degrading	5920523	0.68	5704	3955	[12,29]
*Mycobacterium* *gilvum* Spyr1	FL	Fast	Non-pathogenic	PAH-degrading	5783292	0.68	5434	4038	[13]
*Mycobacterium* sp. JDM601	FHA	Slow	Pathogenic	PAH-non-degrading	4643668	0.68	4398	3162	[30]
*Mycobacterium* *marinum* M, ATCC BAA-535	FHA	Slow	Pathogenic	PAH-non-degrading	6660144	0.66	5501	3717	[31]
*Mycobacterium* *ulcerans* Agy99	FHA	Slow	Pathogenic	PAH-non-degrading	5805761	0.65	4306	2853	[32,33]
*Mycobacterium avium* 104	FHA	Slow	Pathogenic	PAH-non-degrading	5475491	0.69	5305	3504	[34,35]
*Mycobacterium abscessus* CIP 104536	FHA	Fast	Pathogenic	PAH-non-degrading	5090491	0.64	4991	3301	[36]
*Mycobacterium avium* subsp. *paratuberculosis* K-10	FHA	Slow	Pathogenic	PAH-non-degrading	4829781	0.69	4415	3188	[37-39]
*Mycobacterium canettii* CIPT 140010059	FHA	Slow	Pathogenic	PAH-non-degrading	4482059	0.66	3909	2949	[40]
*Mycobacterium tuberculosis* F11 (ExPEC)	FHA	Slow	Pathogenic	PAH-non-degrading	4424435	0.66	4019	2791	[41]
*Mycobacterium tuberculosis* H37Ra	FHA	Slow	Pathogenic	PAH-non-degrading	4419977	0.66	4099	2811	[42-44]
*Mycobacterium tuberculosis* H37Rv (lab strain)	FHA	Slow	Pathogenic	PAH-non-degrading	4411532	0.66	4062	2807	[44]
*Mycobacterium tuberculosis* CCDC5180	FHA	Slow	Pathogenic	PAH-non-degrading	4405981	0.66	3639	2836	[40,45,46]
*Mycobacterium tuberculosis* CDC1551	FHA	Slow	Pathogenic	PAH-non-degrading	4403706	0.66	4300	2716	[47,48]
*Mycobacterium tuberculosis* CCDC5079	FHA	Slow	Pathogenic	PAH-non-degrading	4398812	0.66	3695	2875	[49]
*Mycobacterium tuberculosis* KZN 1435 (MDR)	FHA	Slow	Pathogenic	PAH-non-degrading	4398250	0.66	4107	2799	[25,29]
*Mycobacterium africanum* GM041182	FHA	Slow	Pathogenic	PAH-non-degrading	4389314	0.66	3880	2896	[50,51]
*Mycobacterium bovis* BCG Pasteur 1173P2	FHA	Slow	Pathogenic	PAH-non-degrading	4374522	0.66	4048	2783	[42,43]
*Mycobacterium bovis* BCG Tokyo 172	FHA	Slow	Pathogenic	PAH-non-degrading	4371711	0.66	3996	2776	[52]
*Mycobacterium bovis* AF2122/97	FHA	Slow	Pathogenic	PAH-non-degrading	4345492	0.66	4014	2760	[53]
*Mycobacterium leprae* TN	OI	Slow	Pathogenic	PAH-non-degrading	3268203	0.58	2750	1175	[53-55]
*Mycobacterium leprae* Br4923	OI	Slow	Pathogenic	PAH-non-degrading	3268071	0.58	1654	1173	[56]

^a^FL, free-living; FHA, facultatively-host-associated; OI, obligately intracellular.

### A compendium of phenotypic and genomic changes on the bases of phylogeny, genomic contents, and phenotypes in the genus *Mycobacterium*

**Figure 2:**
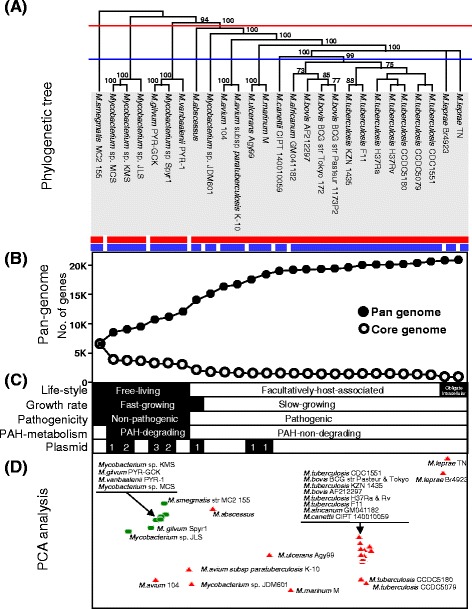
**Clustering and pan**-**genomic change of the 27 fully genome**-**sequenced mycobacteria. (A)** Maximum likelihood tree was constructed by concatenation of the 22 conserved genes in the 27 mycobacteria (available on TreeBASE S16971). The tree was rooted using the free-living *M. smegmatis* MC^2^ 155, with the largest genome size, as an outgroup. The branches were annotated with bootstrap support. **(B)** Effect on the pan- and core genome dynamics found by a progressive addition of genomes based on phylogeny. **(C)** Dichotomous phenotype clustering corresponding to phylogeny for lifestyle, growth rate, pathogenicity, PAH-metabolism, and plasmid content. **(D)** Functional clustering of the 27 mycobacterial genomes based on COG functions. Green circles and red triangles indicate free-living and host-dependent lifestyles, respectively.

In this study, several types of information, including phylogenetic, genomic and phenotypic traits, were integrated to offer a rational comparative clustering of the 27 mycobacterial strains (Table 1), which allowed the following systematic pan-genomic analyses of the evolutionary events in terms of mycobacterial PAH metabolism. Figure 2 shows comprehensive relationships of the 27 mycobacterial strains with respect to phylogeny, genome, and phenotypic traits.

### Phenotypic dichotomy of the well-characterized 27 mycobacterial strains

Classical methods to catalogue mycobacteria are based on convenient phenotypic traits, such as growth rate, pigmentation, pathogenicity, and specific biochemical properties [57]. For the 27 mycobacterial strains, lifestyle (free-living, facultatively host-associated, or obligately intracellular), growth rate (fast- or slow-growing), and pathogenicity (non-pathogenic or pathogenic), together with PAH-degrading ability (PAH-degrading or PAH-non-degrading), all of which are fundamentally heterogeneous general phenotypes with low resolution, were used in dichotomous keys for two-group groupings and comparisons.

Traditionally, a major descriptive division of mycobacteria is related to growth rate: the fast-growers (colonies seen within 7 days) and the slow-growers (colonies not seen within 7 days) [2,3]. Out of the 27 mycobacteria, *M. smegmatis* [25], *M. gilvum* [13], *M. vanbaalenii* [26,27], *M. abscessus* [36] and *Mycobacterium* sp. strains MCS [12,29], KMS [12], and JLS [12] are fast-growers, whereas other mycobacteria, including *M. avium* [34,35], *M. ulcerans* [32,33], *M. marinum* [31], *M. canettii* [40], the *M. tuberculosis* complex (with *M. bovis* and *M. africanum*) [8,16,24,25,29,40-53], and *M. leprae* [37,54-56], are slow-growers. On the basis of pathogenicity, all slow-growing mycobacteria are known to be associated with human or animal diseases, whereas the group of fast-growing mycobacteria has only one important human pathogen, *M. abscessus* [36]. Non-pathogens and fast-growers are free-living (FL), whereas all pathogenic mycobacteria are either host-dependent, facultatively-host-associated (FHA), or obligate intracellular (OI) parasites. All mycobacteria that degrade PAHs have overlapping phenotypic features; they are fast-growing, non-pathogenic and free-living. One or more plasmids are found in many free-living mycobacteria, particularly in the PAH-degraders, and in a few pathogenic FHA mycobacteria, but not in the *M. tuberculosis* complex [16,24,25,29,40-53] or *M. leprae* [37,54-56].

### Phylogeny of the 27 mycobacterial strains

Compared with a 16S rRNA-based tree (data not shown), despite its significant structural similarity, the evolutionary reconstruction based on the concatenation of the sequences of 22 conserved genes (in the core genome of the 27 mycobacterial genomes) substantially enhanced the vertical phylogenetic resolution (Figure 2A, available on TreeBASE S16971). The enhanced discriminatory power was able to segregate the pathogenic and PAH-non-degrading *M. abscessus* from the cluster of PAH-degrading strains in the tree. Therefore, the concatenated sequence-based phylogeny was in congruence with the phenotypic traits, lumping groups of mycobacterial strains together that have common phenotypic characteristics, in terms of lifestyle, growth rate, pathogenic characteristics, and PAH-metabolic capabilities (Figure 2C). The depth of phylogeny could function as a threshold to classify the genus *Mycobacterium* at the degree of phenotypic dichotomy. In the case of growth rate, the fast-growing mycobacteria are more deeply branched to the left of the tree, separated from the slow-growing strains, which form monophyletic clusters with a high intra-cluster relationship (Figure 2A). Here, the fast-growing but pathogenic *M. abscessus* is positioned between the fast-growing PAH-degraders and the slow-growing pathogenic *Mycobacterium* sp. JDM601 (Figure 2A and C). The six well-known PAH-degrading mycobacteria are grouped into an isolated cluster embedding *M. vanbaalenii* PYR-1 (Figure 2A and C). As illustrated in Figure 2A, the distinction between PAH-degraders and PAH-non-degraders can also be easily made on the threshold of the red dashed line. The 27 mycobacteria were clustered into 3 groups (*M. smegmatis*, the six PAH-degraders, and the twenty PAH-non-degrading pathogens), in which members belonging to the same clusters shared the same PAH-degrading metabolic capability.

Overall, the concatenation of the conserved genes enhanced discriminatory resolution of the mycobacterial phylogeny, and despite their polyphyletic and heterogeneous properties, the phenotypes as dichotomous keys for two-group groupings and comparisons function well as a reliable indicator of phylogenetic relationships in the tree, greatly facilitating the phenotypic delineation of clusters. Especially, the combination of the phylogeny and the phenotypic dichotomy provided a global view for the evolutionary process and direction of the genus *Mycobacterium*. Therefore, the integrated use of phenotypic and genotypic (or genomic) traits was essential for better understanding and dissecting of the heterogeneity of the phenotypes at the genomic (or pan-genomic) perspective, which is a key for tracing the evolutionary events.

### Pan-genomic dynamics according to the phylogeny of the 27 mycobacterial strains

The structure and phenotypic behavior of the phylogeny for the genus *Mycobacterium* agree with the proposed model of bacterial evolution: from free-living organisms with larger genomes to host-dependent pathogenic organisms with smaller genomes [58,59]. Based on the observed genome alteration, it could be inferred that the genome dynamics reflected selective environmental forces, which defined the degree of diversity and dynamics of genomes corresponding to phenotypic evolution.

On the basis of the pan-genomic concept [60,61], genome dynamics according to the phylogeny of the 27 mycobacteria were measured to assess the evolutionary scale against which the evolutionary steps led to the contemporary genomes. As shown in Figure 2B and C, the mycobacterial pan-genome shows initially ‘open’ but later ‘closed’ pan-genomic properties. The size of the pan-genome increased rapidly at the beginning from *M. smegmatis* to *M. marinum* via the six PAH-degraders (‘open’ pan-genome added approximately 11,686 new genes), but the number of genes added to the pan-genome was comparatively much smaller with the genomes of pathogenic strains of the *M. africanum*, *M. bovis*, and *M. tuberculosis* clade (‘closed’ pan-genome added only 1,363 genes). On the other hand, the core-genome dropped significantly in size after the addition of the first PAH-degrader *Mycobacterium* sp. MCS (loss of 2,973 genes) and the first pathogen, *M. abscessus* (loss of 1,005 genes). Another noticeable drop was observed in the OI parasite *M. leprae* genome (loss of 425 genes), which is consistent with the phenotypic changes. Interestingly, *M. abscessus*, *M. avium*, *Mycobacterium* sp. JDM601, *M. ulcerans*, and *M. marinum* were scattered away from the stable clusters of the six PAH-degrading mycobacteria and the 12 *M. tuberculosis* complex strains (Figure 2A and D). The mycobacteria (*M. abscessus*, *M. avium*, *Mycobacterium* sp. JDM601, *M. ulcerans*, and *M. marinum*) showed a continuous, rapid pan-genome growth, which was followed by a relatively stable size of core-genome after the transient decline by *M. abscessus*. On the contrary, the 12 *M. tuberculosis* complex strains, with close phylogenetic relationships, showed stable sizes of pan- (only avg. 110 genes increasing) and core-genomes (only avg. 10 genes decreasing), leading to a ‘closed’ pan-genome. The core-genome and pan-genome for all 27 mycobacterial genomes were estimated to be approximately 925 and 20,342 genes, respectively.

In pan-genomic increase, the six PAH-degraders showed an ‘open’ pan-genomic property, a continuous and gradual pan-genome growth, which added approximately 5,320 new genes. The addition of the first *Mycobacterium* sp. MCS to the genome of the PAH-non-degrading *M. smegmatis* contributed 1,870 new genes to the pan-genome. With the five PAH-degrading genomes, the pan-genome grew by an average of 690 (±367) genes every time a new genome was added. On the other hand, after the first *Mycobacterium* sp. MCS caused a significant drop (loss of about 2,973 genes) in the core genome, additional genomes of the PAH-degrading mycobacteria had a minimal effect on the core-genome, which indicates that the core-genome size is relatively static. Since the genomes are similar in size, a continuous and rapid pan-genomic increase while maintaining a stable core-genome indicates a high variability of dispensable (found in one or more but not all genomes) parts of the genomes of PAH-degrading mycobacteria. Overall, the pan-genomic features of the PAH-degraders, the apparent drop of the core-genome in size and the continuous and rapid growth of the pan-genome agree well with their phylogenetic and phenotypic isolation in the genus *Mycobacterium*.

### Functional clustering analysis based on the genomic contents of the 27 mycobacterial strains

To visualize the function-based genomic comparison of the 27 mycobacteria, which evolve through vertical inheritance and horizontal gene transfer (HGT), genomes were clustered based on similar functional profiles. In general, as shown in the principal components analysis (PCA) clustering (Figure 2D), the vertical phylogenetic relevance was well conserved in the cluster structure, based on the genomic clusters of orthologous groups (COG) content distance, and enriched the genome-scale perspective of lateral evolution at a functional level. In addition, the proximity of clustering accounted well for not only the pan-genomic dynamics but also the phenotypic features of the 27 mycobacteria at the genus level. For instance, when clustered by the blue threshold line in the phylogenetic tree (Figure 2A), the clade of 20 PAH-non-degrading pathogenic mycobacteria was further divided into 7 clusters, which provides enhanced discriminatory resolution, reflecting a transitional stage in terms of phenotype and pan-genome evolution. In the functional cluster structure, despite the conserved phenotypic spectrum, six mycobacteria, *M. abscessus*, *M. avium*, *M. ulcerans*, *M. avium* subsp. *paratuberculosis* K-10, *Mycobacterium* sp. JDM601, and *M. marinum*, were dispersed clearly away from the stable clusters of the six PAH-degraders and the *M. tuberculosis* complex and *M. leprae*, the OI parasites (Figure 2D).

Consistent with the phylogenetic affinity and pan-genomic properties, the six PAH-degrading mycobacteria were clustered together, indicating that they share similar functional genomics inventories. Interestingly, despite its significant vertical phylogenetic distance and pan-genomic difference, the PAH-non-degrading *M. smegmatis* was also in close proximity to the cluster of four PAH-degrading mycobacteria (strains KMS, PR-GCK, PYR-1 and MCS). The closer proximity of the PAH-non-degrader to four of the PAH-degraders than to the other PAH-degrader, *Mycobacterium* sp. JLS, indicates a high degree of functional genomics similarity between *M. smegmatis* and the four PAH-degrading strains (Figure 2D) and, as revealed by the following genomic analysis, *M. smegmatis* also had several genes involved in PAH degradation. The observed functional genomics distance suggests that the PAH-degraders share similar genetic and physiological backgrounds for successful microbial metabolism of PAHs, which is apparently an attractive phenotype to mycobacteria in an oligotrophic habitat highly contaminated with PAHs [21].

### Reconstruction of a mycobacterial phenotype network (MPN) for the genus *Mycobacterium*

**Figure 3:**
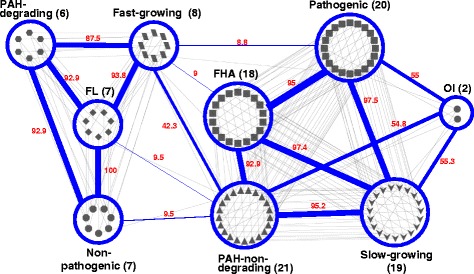
**Mycobacterial** **phenotype network (MPN) of the 27 mycobacteria.** The number of mycobacteria belonging to the phenotype is given in brackets after each name of a phenotype node (blue ellipse). In the MPN, the node shapes of the 27 mycobacterial strains were represented based on the 9-phenotype classification as explained in the text: diamond, FL; hexagon, PAH-degrading; octagon, Non-pathogenic; parallelogram, Fast-growing; rectangle, FHA; triangle, PAH-non-degrading; round rectangle, Pathogenic; v, Slow-growing; ellipse, OI. Together with the numerical connection strength score (in red), the thickness of the connection lines (edge, blue) among the phenotypes indicates the degree of the connection strength. In the MPN, the phenotypes FL and Non-pathogenic show the highest connection strength [CS (Gx, Gy), 100], while Fast-growing (8)—Pathogenic (20), Fast-growing (8)—FHA (18), FL (7)—PAH-non-degrading (21), and Non-pathogenic (7)—PAH-non-degrading (21) show weak connection strength [8.8 < CS (Gx, Gy) < 9.5].

Phenotype networks (PNs) describe the interconnectivity among phenotypic traits. They have been shown to be important in better understanding complex epistatic and pleiotropic behaviors of biological systems from a global perspective [23]. To decipher the genomic epistasis and pleiotropy for mycobacterial phenotypic evolution in terms of PAH degradation, we first reconstructed an MPN and then performed sequential functional pan-genomic comparison, using the phenotype nodes to elucidate emerging properties of the MPN as a whole. The MPN was reconstructed from vertical phenotypic interrelationships as well as horizontal phenotype dichotomy, which accounts for the mutual effects on phenotypic evolution (Figure 3).

The topological structure of the MPN clearly indicates that the mycobacterial phenotypes are linked with scale-free features: (i) preferential linking among phenotype nodes and (ii) a strongly connected hub phenotype node. In the MPN, all phenotype nodes apparently show lifestyle-centric preferential connections, reflecting their phenotypic relationship; three nodes, ‘PAH-degrading’, ‘Fast-growing’, and ‘Non-pathogenic’ are connected to each other with the center at the ‘Free-living (FL)’ node, whereas the other three nodes, ‘Pathogenic’, ‘PAH-non-degrading’, and ‘Slow-growing’ are linked to each other with centers at the ‘facultatively-host-associated (FHA)’ and ‘obligately intracellular (OI)’ nodes (Figure 3). In the MPN, most phenotypes showed strong connectivity, with the exception of the phenotype ‘Fast-Growing’. The phenotype ‘Fast-growing’ is strongly linked to the ‘Free-living (FL)’-centric nodes (‘PAH-degrading’ and ‘Non-pathogenic’) but has a weak connection to the phenotypes, ‘FHA’ and ‘Pathogenic’. The weak connection of the phenotype node ‘Fast-growing’ toward ‘FHA’ and ‘Pathogenic’ caused by *M. abscessus*, which is a fast-growing but facultatively-host-associated pathogenic mycobacterium. Together with *M. abscessus*, medically important fast-growing pathogenic mycobacteria are essentially limited to *M. chelonae* and *M. fortuitum*, which are associated with traumatic and surgical wound infections, skin and soft tissue infections, and pulmonary disease [62]. It should be noted that the weak connection of the node ‘PAH-non-degrading’ with the ‘Fast-growing’ and ‘Non-pathogenic’ nodes resulted from narrow observation caused by the limited number of available mycobacterial genomes. As revealed in the comprehensive topological structure of the MPN, therefore, the phenotype ‘Fast-growing’ shows a transitional phenotypic relationship, bridging the different lifestyle-centric phenotype nodes, which is consistent with its phylogenetic and functional positions.

Another interesting finding was that the phenotype ‘PAH-non-degrading’ is a hub phenotype in the MPN with the highest connection degree of 7 (Figure 3). Consistent with the highest phenotypic compatibility of the hub node, its dichotomy phenotype ‘PAH-degrading’ is a relatively specific and isolated phenotype in the MPN. The ‘PAH-degrading’ node has the lowest connection degree of 3 and an apparently biased connection toward the other two FL-centric phenotypic nodes, ‘Fast-growing’ and ‘Non-pathogenic’. That is, the ‘PAH-degrading’ node has no connection with the phenotypes ‘Pathogenic’ and ‘Slow-growing’, which are the main phenotypic traits of the ‘FHA’ and ‘OI’ mycobacteria. These connection features of the ‘PAH-degrading’ node in the MPN suggest that PAH degradation might be an isolated specific phenotype, limited to a phylogenetically isolated group of mycobacteria.

Overall, together with a useful mycobacterial compendium of phenotypic and genomic properties (Figure 2), the apparent lifestyle-dependent phenotypic connection of the MPN provides global insights into the direction of phenotypic evolution, including PAH degradation, in the genus *Mycobacterium*. In addition, network features of the ‘PAH-degrading’ phenotype in the MPN suggest a relatively low degree of robustness in the face of genetic and environmental changes.

### Pan-genomic perspective of the MPN

**Figure 4:**
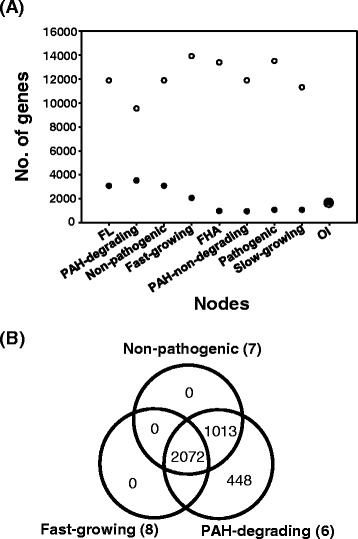
**Comparative pan-genomics based on the MPN. (A)** Pan-genomic comparison (core- and pan-genome) of the phenotype nodes in the MPN. The open and closed circles indicate the number of genes of the pan-genome and core-genome of each phenotypic node, respectively. The pan- (open circle) and core-genome (closed circle) of the phenotype node OI overlapped. **(B)** Venn diagram analysis of the core genomes of the three phenotype nodes in the FL-centric sub-MPN.

The pan-genomic comparison (core- and pan-genomes) of the phenotypic nodes not only further supports the interrelationships and stability of the phenotype nodes in the MPN but also allows better understanding of the relative roles of the core- and dispensable-genomes in phenotype evolution. For quantitative comparison of the phenotypic nodes, we extended the pan-genomic concept to the MPN. As revealed in the pan-genomic comparison (Figure 4A), the ‘Free-living (FL)’-centric phenotype nodes have a relatively large core-genome but a small dispensable-genome compared to the ‘FHA’- and ‘OI’-centric nodes. For instance, the seven sympatric mycobacteria belonging to the ‘FL’ node have a relatively large genome size and core-genome than those of the two allopatric strains in the ‘OI’ node. In addition, there is an association between connectivity of the phenotype nodes and their pan-genomic size, in which the phenotypic nodes with strong connectivity in the MPN show a similar pan-genome size (Figure 4A). The three nodes ‘PAH-degrading’, ‘Free-living (FL)’ and ‘Non-pathogenic’ show a similar pan-genome to core-genome ratio, which are 9,532/3,533 for the ‘PAH-degrading’ node and 11,878/3,085 for the ‘FL’ and ‘Non-pathogenic’ nodes. The ‘Fast-growing’ is an exception to this, having a relatively small core-genome of 2,072 and a large pan-genome of 13,909, which is similar to those of the FHA-centric nodes. The FHA- and OI-centric nodes, ‘PAH-non-degrading’, ‘Pathogenic’, and ‘Slow-growing’, have significantly bigger pan-genomes but smaller core-genome ratios than those of the ‘FL’-centric nodes, which are 13,541/958 genes for the ‘PAH-non-degrading’, 13,541/983 genes for ‘Pathogenic’, and 11,308/1,077 genes for ‘Slow-growing’. As a result, all FHA- and OI-centric phenotypic nodes have relatively small core-genomes but large dispensable genomes, which indicate a high degree of genome diversity of the phenotype node. Most of all, the bigger core-genome size of the ‘FL’-centric nodes than those of the ‘FHA’- and ‘OI’-centric nodes results from both higher numbers of genes per genome and genomic similarity. It suggests that the relatively small core-genome and large pan-genome of the ‘Fast-growing’ node definitely resulted from inclusion of *M. abscessus*, which is distant from other free-living mycobacteria in terms of phylogeny and phenotype.

In the ‘PAH-degrading’ node, the pan-genome contains 9,532 genes, of which 3,533 genes belong to the core-genome that is present in each strain and 5,999 genes belong to the dispensable genome that is absent in one or more strains (Figure 4A). Except for the node OI, the phenotype ‘PAH-degrading’ node shows the smallest pan-genome and dispensable genome and the largest core-genome. This pan-genomic feature clearly suggests a high degree of genomic similarity between members of the PAH-degraders and the core-genome with unique genes, essential for the ‘PAH-degrading’ phenotype. A Venn diagram analysis using the core-genomes of the ‘FL’-centric phenotype nodes revealed that the ‘PAH-degrading’ node has several hundred unique genes (Figure 4B). Out of 448 unique genes, about 34 genes encode the catabolic enzymes involved in RCP (18, 53%), SCP (12, 35%), and CAP (4, 12%) that are responsible for PAH metabolism in *M. vanbaalenii* strain PYR-1, whereas about 415 genes encode enzymes for diverse cellular functions, such as 143 genes (35%) for cellular metabolism (MET), 44 genes (11%) for information storage and processing (ISP), 57 genes (14%) for cellular processes and signaling (CPS), and 90 genes (22%) that are poorly characterized. Consistent with the pan-genomic properties of the ‘PAH-degrading’ node, its dichotomy phenotype node, ‘PAH-non-degrading”, shows the smallest core-genome of 958 genes and largest pan-genome of 13,541 genes. The pan-genomic features of the ‘PAH-degrading’ node are consistent with the monophyletic affinity in phylogeny and the biased connection preference to the phenotype nodes (‘FL’, ‘Non-pathogenic’, and ‘Fast-growing’ nodes) with similar pan-genomic sizes in the MPN.

### Functional pan-genomic view of the six PAH-degraders with PAH-degrading phenotype in the MPN

**Figure 5:**
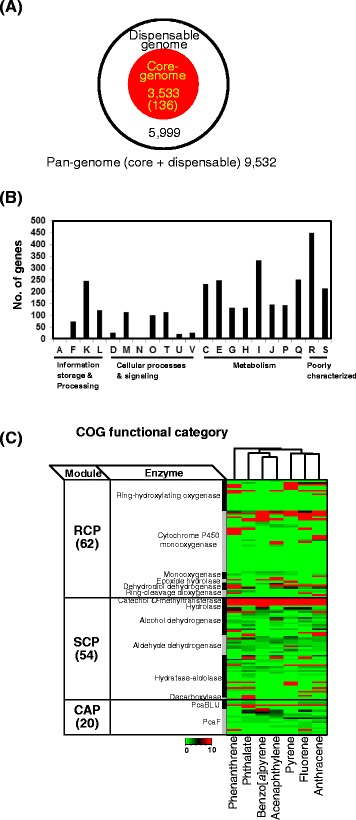
**Systematic genome analyses of the six PAH-degrading mycobacteria. (A)** Pan-genome of the six PAH-degrading mycobacteria. **(B)** Functional classification of the 3,533 core-genome based on COG category. **(C)** Classification of the 136 catabolic enzymes based on functional module and enzyme function in PAH-MN. Proteomic expression patterns under different aromatic hydrocarbon treatments are also shown.

For functional pan-genomic understanding of the PAH-degrading phenotype, we investigated the pan-genome of the six PAH-degrading mycobacteria in the ‘PAH-degrading’ node based on the previous proteomic data from *M. vanbaalenii* PYR-1 [9]. As shown in Figure 5A and Additional file 1: Table S1-S5, we categorized the pan-genome of 9,532 genes from the six strains, among which 3,533 genes were considered to be core genome and 5,999 genes were dispensable genome. The core-genome accounted for 37% of the pan-genome and, as shown in functional analysis, shows a distribution across all COG categories (Figure 5B; Additional file 1: Tables S1 and S2).

Among the 3,533 core genes, 136 genes were tentatively identified to be involved in the degradation of aromatic hydrocarbons (Additional file 1: Table S3). According to the functional module proposed in the PAH-MN in *M. vanbaalenii* PYR-1 [9], about 62 genes, involved in the functional module RCP, made up the largest portion of the list of 136 genes, which is followed by 54 and 20 genes, involved in SCP and CAP, respectively (Figure 5C; Additional file 1: Table S4). Especially, as shown in the proteome data (Figure 5C), about 113 genes out of the 136 genes are expressed as proteins involved in PAH degradation pathways (Additional file 1: Table S5) and the expression of these proteins is apparently PAH-substrate-regulated. Therefore, this functional genomics observation not only clearly indicates that the proteins of the core genome function for PAH-metabolism but also provides some insights into the genetic requirements and metabolic potential in terms of PAH degradation; they share similar genetic repertoires and PAH metabolic spectra.

### Contribution of HGT events to the PAH-degrading phenotype

**Figure 6:**
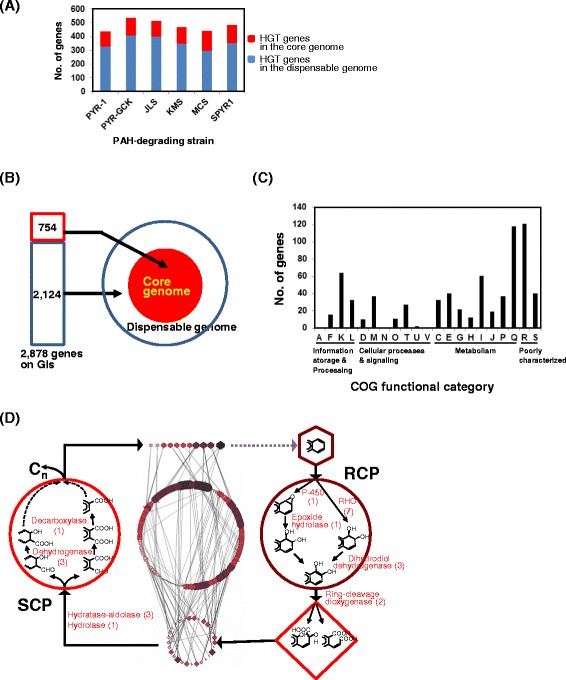
**Genomic islands (GIs, large-genomic regions that contain multiple genes of probable horizontal origin) analysis of the PAH-degrading mycobacteria. (A)** Comparison of the GIs from the PAH-degrading mycobacteria, depending upon core and dispensable genes. **(B)** HGT contribution of the 2,878 GI-located genes to the pan-genome of PAH-degrading mycobacteria. **(C)** Functional distribution of the 754 horizontally transferred genes to the core-genome based on COG. **(D)** Functional annotation of the horizontally transferred PAH-catabolic genes with respect to the general scheme of PAH metabolism proposed for *M. vanbaalenii* PYR-1. Only the 22 genes involved in RCP and SCP are shown. Please see reference [9] for a more detailed explanation of the general scheme of mycobacterial PAH metabolism.

Most free-living mycobacteria are naturally competent, that is, they can take up genetic material from the environment and recombine it into their chromosome [63]. Analysis of the genome sequences of the PAH-degrading mycobacteria revealed many horizontally acquired DNA regions (genomic islands [GIs]) interspersed in their genomes. These GIs are mostly larger than 4 kb in length (4 ~ 20 kb in size), with average numbers being 35.8 (±7.5) per genome (data not shown). The number of HGT genes identified in the PAH-degrading mycobacteria ranged from 437, in *M. vanbaalenii* PYR-1, to 536, in *M. gilvum* PYR-GCK (average of 480 ± 40), and is 2,878 in total (Figure 6A; Additional file 2: Table S6). Among these HGT genes, we identified 754 and 2,124 genes that contribute to the core-genome (a total of 3,533 genes) and the dispensable genome (a total of 5,999 genes), respectively (Figure 6B; Additional file 2: Tables S7-8). Interestingly, the contribution of the horizontally transferred 754 genes to the 5 COG cellular metabolism categories, C, E, F, G, H, and I, was 339 genes (53%) (Figure 6C), which is apparently higher than that seen in the genomes of PAH-degrading mycobacteria (around 30%). It indicates that GIs contribute to the metabolic proficiency of these organisms, including the ability to degrade PAHs.

Considering only PAH degradation, we identified as horizontally transferred 29 genes, which are enough to cover all the enzymatic reactions in the general scheme of PAH degradation proposed for *M. vanbaalenii* PYR-1 (Figure 6D; Additional file 2: Tables S9-10) [9]. Many PAH-catabolic genes identified on GIs were also located on the plasmids of PAH-degrading mycobacteria, such as *Mycobacterium* spp. MCS and KMS, which were isolated from geographically different locations. Comparison of GIs and PAH-catabolic genes among these PAH degraders showed conservation of ~150-kb catabolic gene clusters, with slightly different genetic configurations (data not shown). Together with the pan-genomic evidence, it strongly demonstrates that most PAH-degrading strains share the same genetic sources and pathway evolution by patchwork assembly (assembly of existing pathways in new combinations), which has been systematically evaluated as highly successful [9]. It appears that the HGT events of the highly conserved PAH-degrading gene clusters could provide practical benefits: (i) overcoming evolutionary selective pressure, (ii) acceleration of the buildup speed, and (iii) metabolic maximization from the limited genetic modules [9,64].

### Unique genes in PAH-degrading core-genome but not the pan-genome of PAH-non-degraders

**Figure 7:**
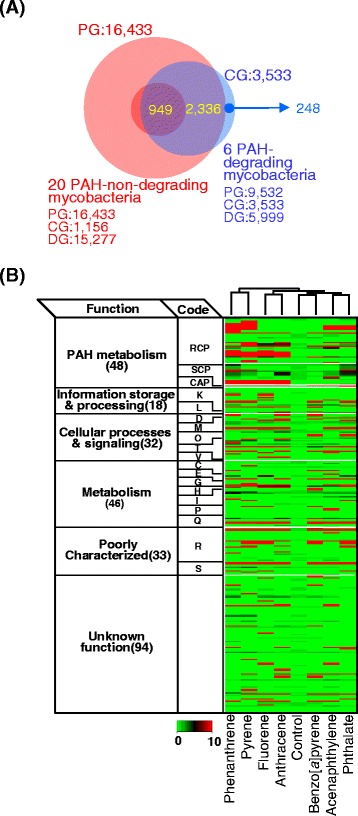
**Identification of the genes found only in the six PAH-degrading mycobacteria. (A)** Comparison of the core genome of the six PAH-degrading mycobacteria and pan-genome of the 20 PAH-non-degrading mycobacteria. **(B)** Functional classification and their regulation of the unique genes in the core genome based on the COG functional category. Please see reference [9] for the detailed protein expression information.

Under dynamic HGT events, the PAH-degrading phenotype might be equally open to all mycobacteria. As expected from the pan-genomic view, however, the ‘PAH-degrading’ HGT events do not have the same metabolic effects on all competent mycobacteria, because of several types of influences, including environment and stochastic, molecular or epigenetic variation among individuals [58,65,66]. However, other major influences on HGT events could be the genetic backgrounds of competent mycobacteria. To understand the unique genetic backgrounds related to the PAH-degrading phenotype, a comparative analysis of the core-genome of the ‘PAH-degrading’ node and the pan-genome of the ‘PAH-non-degrading’ node was conducted. It led us to find unique genes, which might be directly or indirectly related to PAH metabolism, in all PAH-degrading mycobacteria. As shown in Figure 7A and Additional file 3: Tables S11-12, about 248 unique genes were identified only in the core-genome of the PAH-degrading mycobacterial genomes. From the list of 248 unique genes, 154 genes (62%) were categorized based on COG function or functional modules in the PAH-MN, among which 35 genes were assigned to RCP (23 genes), SCP (10 genes), and CAP (2 genes) for PAH metabolism [9]. The other 119 genes were assigned to other cellular functions (Figure 7B; Additional file 3: Tables S11-12). Interestingly, 46 genes (19%) of the unique gene pool were identified as horizontally transferred. When analyzed in terms of protein expression, 146 (59%) genes were expressed under all experimental conditions, with 28 (61% of 46 genes) horizontally transferred genes.

Out of the 23 proteins assigned to the RCP functional module in the PAH-MN, 12 genes encode oxygenase components for six ring-hydroxylating oxygenase (RHO) enzyme systems [9,67]. Six of the 12 genes encode the RHO components, NidAB, NidA3B3, and PdoA2B2, which belong to a type V RHO system, while the other 6 genes encode RHO enzymes belonging to a type X RHO system, based on Kweon’s RHO classification [67-69]. Notably, these 3 RHOs, NidAB, NidA3B3, and PdoA2B2, have been shown to be the main enzymes involved in the initial oxidation of HMW PAHs, such as pyrene and fluoranthene, and LMW PAHs, such as phenanthrene, anthracene, and fluorene [5-8,14,15,18,19,67,68,70,71].

Overall, it appears that the conserved unique gene pool contained only in the PAH-degraders was obtained mainly by HGT events and plays crucial roles in the PAH-degrading phenotype, either directly by the PAH-degrading enzymes, such as the three type V RHO systems in the RCP functional module, or indirectly by the 120 proteins with versatile cellular functions. As revealed in the following gene evolutionary analysis using the Count software [72], considering the significant contribution of HGT events to the conserved unique genes, the uniqueness of the PAH-degradation genes identified only in the PAH-degraders might be explained by gene loss during evolution of the PAH-degrading phenotype under selection. When compared with the core-genome of the PAH-degraders, the unique gene pool also indicates that PAH-non-degraders also have a significant number of genes directly involved in PAH metabolism, which might reveal how closely the PAH-degraders are related to PAH-non-degraders.

### The presence, organization, and dynamics of PAH-degrading genes in the genus *Mycobacterium*

**Figure 8:**
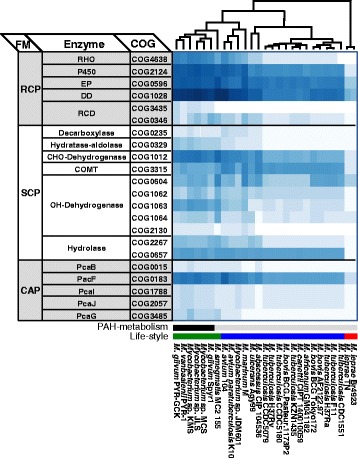
**Distribution of PAH-degrading genes across the genus** ***Mycobacterium*** **.** The genes involved in the PAH-MN were classified based on functional modules and COG functional categories. In the cluster analysis of the heat map, the presence/absence pattern of the PHA-degrading genes shows apparent relationships with the PAH-degrading abilities and lifestyles of the 27 mycobacteria. The six PAH-degraders were grouped together in the left, forming a cluster separated from PAH-non-degraders.

**Figure 9:**
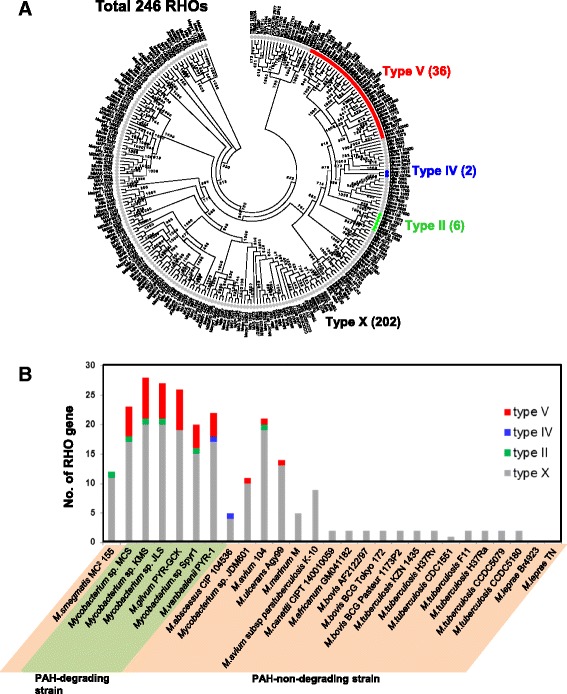
**RHO-centric perspective of the 27 mycobacteria. (A)** Classification of the 246 RHO genes identified from the 27 mycobacteria based on Kweon’s classification [67,69] and **(B)** their genomic distribution. Among the classified RHO genes, the type V RHO system was the most dominant and concentrated in the six PAH-degrading mycobacterial genomes (strains MCS, KMS, JLS, PYR-GCK, Spyr1, and PYR-1). The *M. tuberculosis* complex and the two OI strains, Br4923 and TN, have no genes for type V RHO system.

**Figure 10:**
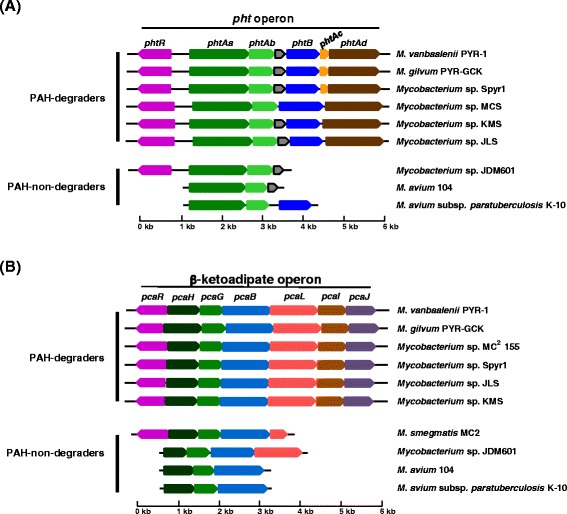
**Comparison of the phthalate operon (A) and the β-ketoadipate operon (B) in the genus** ***Mycobacterium*** **including the six PAH-degraders and the PAH-non-degraders** **.** The pht and β-ketoadipate operons, which link the peripheral pathways (RCPs and SCPs) to the TCA cycle in the PAH-MN, are conserved in the PAH-degraders, whereas some of the PAH-non-degraders, including strains JDM601, 104, and K-10, have a broken operon structure.

**Figure 11:**
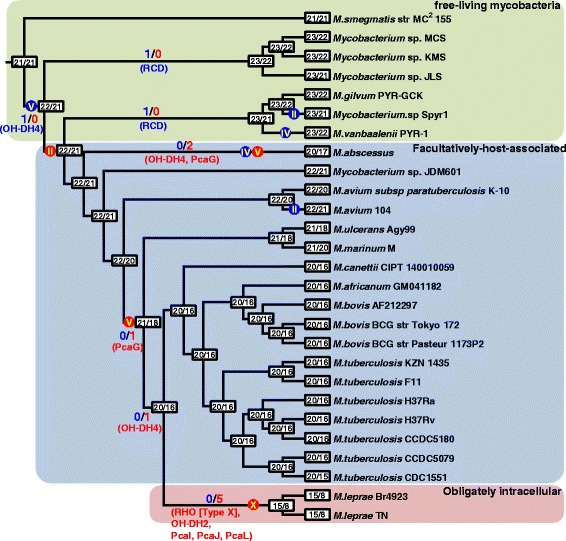
**Gain and loss of PAH-degrading genes in the genus** ***Mycobacterium*** **.** Boxes on nodes and tips for the phylogeny show numbers of the PAH-degrading gene groups and numbers of the groups with more than one member at least, respectively. Numbers and dots on branches indicate the gains and losses of twenty three PAH-degrading gene groups and four types of RHO groups, respectively, with blue indicating an overall gain and red an overall loss. Roman numerals in the dots indicate type (types II, IV, V and X) of the RHO systems based on Kweon’s classification [67,69]. The 27 mycobacteria are color shaded according to their lifestyle: green, free-living; blue, facultatively-host-associated; red, obligately intracellular. OH-DH4, alcohol dehydrogenase 4; RCD, ring-cleavage dioxygenase; PcaG, the alpha subunit of protocatechuate 3,4-dioxygenase; RHO, ring-hydroxylating oxygenase; OH-DH2, alcohol dehydrogenase 2; PcaI, 3-oxoadipate CoA-transferase subunit A; PcaJ, 3-oxoadipate CoA-transferase subunit B; PcaL, 3-oxoadipate enol-lactone hydrolase/4-carboxymuconolactone decarboxylase.

To explain the PAH-degrading phenotype and its evolution in the genus *Mycobacterium*, the presence (Figures 8 and 9), organization (Figure 10), and dynamics (gain/loss, Figure 11) of the PAH-degrading genes, essential for the complete metabolism of PAHs, were analyzed. To address the distribution of PAH-degrading genes and capabilities throughout the genome of mycobacteria, the PAH-degrading genes based on functional modules of the PAH-MN were surveyed [9]. Interestingly, as revealed in the analysis for the unique genes (Figure 7), most putative genes for PAH-degradation are widespread in the genus *Mycobacterium*. The PAH-degrading genes identified were between 37 (*M. leprae* Br4923) and 425 genes (*Mycobacterium* sp. JLS) from the genomes of the 27 mycobacteria (Additional file 4: Table S13). Although overall distribution of the catabolic genes among the mycobacteria is variable, the clustering results clearly show a correlation between the gene profiles and the PAH-degrading ability. As shown in the overall view of the heat map (Figure 8), the six PAH-degraders were clustered together to the left, separated from the “PAH-non-degrading” strains, suggesting genomic differences in terms of PAH-degradation gene distribution. In addition, there is an apparent gradual decrease in the number of genes during evolution from ‘FL’ to ‘OI’ via ‘FHA’ mycobacteria, with several genes showing apparent gain/loss patterns according to PAH-degrading ability (Figures 8, 9, 10, and 11).

In the PAH-MN, the functional module RCP, producing catechols and ring-cleavage products from PAH substrates for the following SCP or CAP, is a crucial process that determines substrate range and pathways [9]. A set of enzymes, including ring-hydroxylating oxygenases (RHOs), cytochrome P450 monooxygenases (CYPs), epoxide hydrolases, cis-dihydrodiol dehydrogenases, and ring-cleavage dioxygenases (RCDs), is responsible for RCP [9]. Among them, two groups of oxygenases, CYPs and RHOs, are known to initiate mycobacterial PAH degradation by ring mono- or di-hydroxylation to form trans- and cis-dihydrodiols, respectively [5,8,9,14,15,70,71,73]. In the RHOs, which are the main oxygenases for initial PAH oxidation, genome analysis of the 27 mycobacteria predicted the existence of a total of 246 genes encoding this RHO enzyme, with a range between 0 in *M. leprae* and 28 in *Mycobacterium* sp. KMS (Figure 9; Additional file 5: Table S14). As illustrated in Figure 9A, out of the 246 RHO enzymes based on Kweon’s classification [67,69], 44 RHO enzymes were assigned to type II (6 RHOs), type IV (2 RHOs), and type V (36 RHOs), and the rest (202 enzymes) were assigned to type X (unclassified RHO) [67,69]. The 36 type V RHO systems are dominant and mostly concentrated in the PAH-degraders, which normally have at least four or more type V RHO systems (Figure 9B). In particular, 29 type V RHOs out of 32 are only present in the PAH-degraders (Figure 9A and B). As revealed by the gene gain/loss analysis (Figure 11), the fact that the three type V RHOs, two Nid (*nidAB* and *nidA3B3*) systems and one Pdo (*pdoA2B2*) system, are consistently present (gained) in the PAH-utilizing mycobacteria and absent (lost) in PAH-non-degraders, further supports their functional importance in PAH metabolism [6,7,9,15,70,71]. Interestingly, together with the 3 type V RHOs, another type V RHO, a Pht system (*phtAaAb*), encoding a phthalate dioxygenase, is also consistently present in the six PAH-degraders as well as in several PAH-non-degraders, such as *Mycobacterium* sp. JDM601, *M. avium* 104, and *M. avium* subsp. *paratuberculosis* K-10 (Figures 9 and 10A). A comparison of the operons containing the Pht system, however, shows that the *pht* operons from the PAH-non-degraders do not have two genes, *phtAc* and *phtAd*, that encode electron transfer components (ETCs), a [3Fe-4S]-type ferredoxin and a GR-type reductase, respectively (Figure 10A). The two genes *phtAc* and *phtAd* comprise a typical type V ETC, which is functionally compatible with type V RHO systems [9,67,68]. The absence of the type V ETC suggests that this incomplete Pht system could not function in the hydroxylation of phthalate, which is one of the main hub intermediates in the PAH-MN of *M. vanbaalenii* [8,9]. Furthermore, the significant numerical imbalance between oxygenase components and ETCs, the functional loss of type V ETC, which has a high degree of compatibility, most likely hinders PAH degradation in these PAH-non-degraders. In the genome of *M. vanbaalenii*, 21 genes were identified to encode large subunit components of RHOs, whereas only one copy of the type V ETC was identified [67,68]. Interestingly, RHO enzymes also gradually decrease in numbers during evolution from ‘FL’ to ‘OI’ via ‘FHA’ mycobacteria (Figures 9 and 11). In contrast, the number of CYPs varies among 27 mycobacterial strains, which means no correlation with PAH-degrading ability of the strains (Figure 8). However, although few of their physiological roles are known, previous studies have proved the involvement of CYP enzymes in the initial oxidation of PAHs as an alternative PAH oxygenation reaction [73]. Among the genes involved in RCP, the gene encoding RCDs (COG3435) showed the lowest gene copy number and only the six PAH-degraders had this RCD gene (Figure 8).

SCP includes the steps that are responsible for hydrolysis of the α-keto side chain of the ring-cleaved compounds, forming biological precursors, such as pyruvate, and, at the same time, metabolites with an aldehyde group, which should be removed during oxidation/decarboxylation in the subsequent reaction [9]. As shown in Figure 8, functionally diverse enzymes are included in this functional module. Generally these enzymes show a significant numerical abundance with minor exceptions, enabling SCP to accept an enormous range of ring-cleavage compounds during PAH metabolism [8,9,18,19]. Two enzymes, the decarboxylase (COG0235) and alcohol dehydrogenase (COG2130), are the exceptions to this observation; only one or two copies have been found in the genomes of PAH-degraders and a few PAH-non-degraders (Figure 8) [9]. Hence, despite these two enzymes’ low gene redundancy, their practical functional contribution to diverse substrates with different sizes and architecture in the PAH-MN accounts for their substrate diversity and relaxed regulation [6,7,9,68]. Interestingly, together with an alcohol dehydrogenase (COG1062, OH-DH2), the alcohol dehydrogenase (COG2130, OH-DH4) has been lost during evolution from ‘FL’ to ‘FHA’ or ‘OI’ mycobacteria (Figure 11). On the contrary, genes encoding catechol-*O*-methyltransferase (COMT; EC 2.1.1.6, COG3315), which plays an important role in the detoxification of catechols in PAH metabolism [18,19], are more abundant in the genomes of PAH-non-degraders (Figure 8), suggesting that some other different functions are probably involved in pathogenicity in the pathogenic mycobacteria (Figure 8). Most enzymes belonging to SCP, including the decarboxylase, are expressed constitutively, which suggests that their regulation is more relaxed than those belonging to the RCP (Figures 5C and 7B).

The highly conserved catabolic genes responsible for RCP and SCP in the PAH-degraders clearly indicate that all six PAH-degraders should convert a broad range of PAHs into phthalate, which is the main hub node with an in-degree of 7 and out-degree of 3 in the PAH-MN from *M. vanbaalenii* PYR-1 [9]. In addition, a synteny block for the β-ketoadipate pathway in the PAH-degraders is conserved (Figure 10B), which further suggests that all outgoing metabolic routes from phthalate could be channeled into the β-ketoadipate pathway via protocatechuate. Hence, the pca gene cluster for the β-ketoadipate pathway inevitably functions for CAP in mycobacterial PAH metabolism (Figures 5C, 7B, 8, and 10B).

A closer examination of the pca clusters from the members of PAH-degraders and PAH-non-degraders provides additional evidence not only for a pivotal role of HGT but also for evolutionary dynamics of operon structures in the evolution of the PAH-degrading phenotype at the genus level (Figure 10B). Despite the benefits conferred by coregulation [74], the *pca* operon is not stable in composition over evolutionary time, as shown in Figure 10B. The *pcaHGBLIJ* genes seem to be resistant for operon breakage in all PAH-degraders, although the gene *pcaF*, encoding β-ketoadipyl-CoA thiolase, the last step enzyme to transform β-ketoadipyl-CoA to succinyl-CoA and acetyl-CoA in the β-ketoadipate pathway [9,75], does not reside in the *pca* cluster. In the PAH-non-degraders *Mycobacterium* sp. JDM601, *M. avium* 104, and *M. avium* subsp. *paratuberculosis* K-10, these operons are drastically reduced, having lost several genes, such as *pcaL,pcaI,pcaJ,* and *pcaF* (Figure 10B). However, although several pca genes are found in the genomes of other PAH-non-degraders, they are completely dispersed (Figures 8 and 10B). Especially, the two OI strains Br4923 and TN have completely lost *pcaI*, *pcaJ,* and *pcaL* (Figure 11). Therefore, stability of the pca operon structure is strongly correlated with the PAH-degrading ability of the genome, functioning as a useful indicator in the assessment of genome evolution in terms of the PAH-degrading phenotype.

### A possible evolutionary path of the PAH-degrading phenotype in the genus *Mycobacterium*

**Figure 12:**
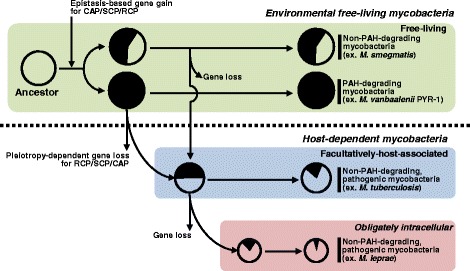
**A plausible evolutionary pathway of the PAH-degrading mycobacterial group.** The scheme is based on the gene gain and loss of PAH-degrading genes and the genomic information (genome and pan-genomes). The size of circles indicates the ratio of the relative size of the genomes. The complete black circles indicate a mycobacterium with the complete gene set for PAH-degradation, while partially blacked circles indicate PAH-non-degrading mycobacteria with incomplete PAH-degrading genes. More filled circles mean more PAH-degrading genes in the genome. Color shading follows Figure 11. Note that all PAH-degrading mycobacteria have larger genome size and more HGT events than PAH-non-degrading mycobacteria, which have some PAH-degrading genes but not a complete set of genes. Especially, OI mycobacteria have the smallest size in terms of genome and the number of PAH-degrading genes. Under strong natural selection, the gene gain/loss by HGT/deletion events hypothesize an epistasis-based birth (from CAP to RCP via SCP) and pleiotropy-dependent death (from RCP and CAP to SCP), for PAH metabolism in the genus *Mycobacterium*.

An integrated view of phylogeny, functional pan-genome, and MPN suggests that mycobacterial genomic dynamics has been communicated to the PAH-degrading phenotype according to its lifestyle. In the genus *Mycobacterium*, PAH-metabolism might be an attractive backup phenotype for alternative carbon-energy sources to free-living mycobacteria, but it seems to have been regarded as dispensable by the host-dependent species. In addition, due to PAH metabolic ambiguity (as nutrient or toxicant), its evolution might not have been simple or even random. The observed gene gain/loss patterns by HGT and deletion hypothesize a possible evolutionary path in which mycobacteria have obtained and preserved the extra metabolic capability while free-living, but during evolution towards the host-dependent life lost this dispensable capability (Figure 12).

### Epistasis-based origin of the mycobacterial PAH-degrading phenotype

Genomic evidence from the 27 fully-sequenced mycobacteria strongly suggests extensive HGT events of the genetic materials related to PAH metabolism in the environment. Furthermore, the genus *Mycobacterium* originally had several metabolic and physiological selective advantages along with this new metabolic phenotype: (i) high specific affinity for PAH substrates, (ii) cell surface hydrophobicity and attachment ability, and (iii) formation of biofilms on PAH particles [4,5,14,15]. Despite such selective advantages, however, the energy required to acquire a PAH-degrading phenotype seems excessively high. For example, for the four ring-structure pyrene to be completely degraded in the PAH-MN, the genomic HGT evidence in this study and the previously proposed evolution hypothesis of the metabolic network suggest that at least 5 different HGT events are required, which should successfully provide the building blocks for a patchwork assembly to cover the entire 27 metabolic steps [6,7]. Actually, since the HGT events can in principle occur in any order, there are 5! = 120 HGT trajectories linking the gene clusters. However, toxic effects of the uncontrolled intermediates, such as o-quinones [18], might not regard all trajectories equivalently. Therefore, productive epistatic interactions of the building blocks should have a strong selection impact on the evolutionary trajectories. Consistent with this opinion, the conserved genetic sources with similar gene cluster structures in the core-genome of the PAH-degrading mycobacteria suggest overlapping evolutionary events and evolutionary trajectories for the PAH-degrading phenotype. Under strong natural selection towards minimized side effects, impacts of the epistatic interaction of the genetic sources might restrict the possible evolutionary trajectories for mycobacteria to be able to obtain the PAH metabolic ability. Hence, all the successful evolutionary trajectories were able to satisfy the functionally reversed growth of the PAH-MN: a patchwork assembly with a growth direction from CAP to RCP via SCP, essential to minimize the toxic effects of the activated PAH intermediates, which are produced in the RCP [9] (Figures 11 and 12).

### Pleiotropy-dependent abandonment of the mycobacterial PAH-degrading phenotype

Despite a high degree of technical difficulty in obtaining a PAH-degrading ability, it could be an attractive phenotype for free-living mycobacteria in terms of nutritional competition in oligotrophic environmental niches [5,20]. On the other hand, the PAH-degrading phenotype might be unnecessary to host-dependent mycobacteria, which do not obtain nutritional benefits from PAH substrates. For example, *M. tuberculosis,* an intracellular pathogen, uses fatty acids and cholesterol from its host [76]. Interestingly, as revealed in the gene gain/loss analysis (Figure 11), there is a clear negative correlation between the size of genetic remnants related to PAH metabolism and the evolutionary time toward the host-dependent life, which suggests the basic evolutionary concept—initially adopted but later abandoned—for the PAH-degrading phenotype in the genus *Mycobacterium* (Figure 12). The instability of the operon structures remaining in the genomes of the host-dependent mycobacteria and the lack of certain genes for PAH catabolic function suggest abandonment of the PAH-degrading phenotype in the evolution towards a host-dependent lifestyle. Because PAHs are absent in the biological hosts, it is likely that this ability has been lost during the host-dependent evolution.

We observed the early events in the disassembly of the PAH-MN in the FHA PAH-non-degrading mycobacterial genomes (*Mycobacterium* sp. JDM601, *M. avium* 104, and *M. avium* subsp. *paratuberculosis* K-10), which are located in the middle of the free-living PAH-degraders and the FHA *M. tuberculosis* complex, while there was a rare opportunity to observe the genetic evidence from the free-living PAH-degrading mycobacteria (Figures 10 and 11). As shown in Figure 10, the operons having genes involved in the phthalate and β-ketoadipate pathways, respectively, according to the PAH-metabolic ability showed different stability; the two operons are protected from breakage in the PAH-degrading strains but are broken in the PAH-non-degrading mycobacterial genomes.

**Figure 13:**
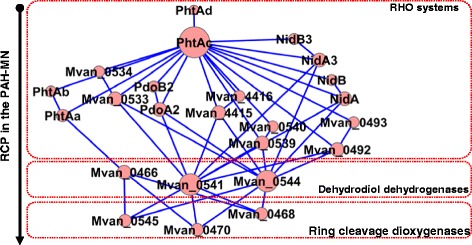
**Scale-free functional interaction network of the enzymes responsible for RCP in the PAH-MN** **.** The functional network was reconstructed based on the enzymes involved in the RCP in PAH-MN, which are expressed as proteins and exist in the core-genome of the PAH-degraders (that is, selected by functional pan-genomics). In the network, the size of nodes is based on the degree of connectivity of the nodes. The main hub node, PhtAc, which shows the highest connectivity with an in-degree of 1 and out-degree of 16, is shown as the biggest node in size. Cytoscape was used for visualizing and analyzing networks in this study [77].

Like the origin of PAH-MN discussed above, the same strength of natural selection minimizing toxic side effects might also have restricted the abandonment trajectories in the evolutionary process for disuse of the dispensable metabolic capability in the host-dependent mycobacteria. Evolutionary rational endeavors seem to have participated in the disassembly process to follow the effective abandonable trajectories to minimize any possible side-effects and to save time and energy. In this respect, the effects of genetic perturbation on functional robustness—correlation between functional redundancy and negative epistasis—from the perspective of quantity and quality in the metabolism of PAHs provide clues to understand the plausible abandonment trajectories. Although the PAH-degrading phenotype is functionally robust to genetic perturbation, the robustness depends mainly on perturbation location in the PAH-MN. The funnel-like topology of the MN is intimately related to its behavior and also to its functional robustness toward genetic perturbation, in which many peripheral pathways converge to the β-ketoadipate pathway with the same input and output diameters in its function, which is linked to the TCA cycle [9]. In general, effects of the functional perturbation on the steps, with extremely low genetic and functional redundancy, propagate across the entire PAH-MN, creating many possible quantitative metabolic damages. In this respect, as revealed in the pan-genomic and RHO profile comparison, the host-dependent mycobacteria seem to have followed the effective abandonment trajectories, which initially tried to have the RHO systems disabled from the RCP modules, responsible mainly for toxic PAH intermediates. However, from the negative epistatic interaction view, RHO systems in the PAH-MN are not good target genes for effective abandonment trajectories, considering their extremely high genetic and functional redundancy. Surprisingly, the host-dependent mycobacteria seem to find the best solution for this problematic requirement from the common feature of mycobacterial RHO systems, which is numerical imbalance between the two functional groups, an oxygenase and an ETC [9,67,68]. As shown in Figure 13, the distinct numerical imbalance between oxygenase components and an ETC clearly indicates that a gene encoding the ETC has strong functionally pleiotropic effects. Based on these observations, the practical metabolic effects of the gene loss for a type V ETC could be significant and effective. Especially, considering their responsibility for the initial ring-hydroxylation of PAH substrates in diverse RCP functional modules (Figure 13), which produce activated toxic intermediates, removal of the RHO function by loss of the strong pleiotropic gene could be effective and time-saving, as observed in the metabolic and proteomic data of PAH-MN [9]. In addition, the three type V RHO enzymes, two Nid systems and a Pdo system responsible mainly for the initial oxygenation of diverse PAH substrates are absent in all PAH-non-degraders. Together with these endeavors, the PAH-non-degraders have kept the enzymes responsible for detoxification. For example, more genes encoding COMTs, involved in the detoxification of toxic PAH intermediates, exist in the PAH-non-degraders (Figure 8).

Taken together, these systematic observations offer strong support for the hypothesis that natural selection for maximized metabolic benefits and minimized side effects of the uncontrolled PAH intermediates has restricted the evolutionary trajectories of mycobacterial PAH metabolism. The evolutionary behavior might take full advantage of the pleiotropic and epistatic effects of gene gain/loss, allowing mycobacteria to follow the few successful trajectories, which satisfy the functional and toxicological requirements in the evolution of PAH metabolism in the genus *Mycobacterium*.

## Conclusions

Taking the whole picture into account, mycobacterial PAH metabolism at the genus level might be an excellent phenotypic model to bridge genomic dynamics and phenotypic evolution, which could address a series of questions for a predictable direction and plausible trajectories for mycobacterial evolution. The reasonable factors and their practical advantages include the following: (i) the metabolic ambiguity of PAHs as carbon and energy sources or toxicants, which might have directed evolutionary responses to maximize the nutritional benefits and minimize the possible side-effects, enhancing opportunities to observe the apparently selective choice of evolutionary trajectories; (ii) the availability (ubiquity) of PAHs in the environment but not in the biological hosts, which might be an apparent selective pressure on its evolution; (iii) the flood of genome sequences and phenotypic information across strains from free-living to obligate intracellular parasites, together with the complete metabolic and genetic knowledge for mycobacterial PAH metabolism, which allow synergistic analysis of phylogenic, phenotypic, and genomic information going into the structure, behavior, and evolution of PAH metabolism.

In this study, we have discovered a wealth of evidence relating mycobacterial evolution to PAH metabolism. This evidence comes in a useful mycobacterial compendium of phenotypic and genomic changes, focusing on the PAH-degrading phenotype with a functional pan-genomic perspective of the evolutionary events and environmental challenges. The compendium enabled us to understand the relation between genomic dynamics and the corresponding phenotypic effects, which helped us to follow a few plausible evolutionary paths to fitter phenotypes. When selection acts on evolution of PAH metabolism, only a small fraction of evolutionary trajectories are likely to be observed, owing mainly to a combination of the ambiguous metabolic effects of PAHs and the corresponding pleiotropy- and epistasis-dependent evolutionary behavior. Systematic functional pan-genomic comparison allows us to follow largely identical evolutionary trajectories, which substantially enriches our understanding of the pleiotropic and epistatic evolutionary process of PAH metabolism at the genus level. Evolutionary constraints on the trajectories seen in the PAH-degrading phenotype are likely to apply to the evolution of other phenotypes in the genus Mycobacterium, which is essential to better characterize mycobacterial communities for medical or bioengineering applications.

## Methods

### Genome sequences and proteomic data sets

In this study, we used only complete genome sequences with well-organized phenotypic and genomic information (Table 1) for reasonable network and functional pan-genomic analysis without numerical bias in terms of mycobacterial phenotypes. All genome sequences were retrieved from the NCBI (http://www.ncbi.nlm.nih.gov/genome/browse/) or IMG/JGI (https://img.jgi.doe.gov/cgi-bin/w/main.cgi). The NCBI gbk and ptt (a tab delimited file containing a list of all the proteins for the genome) files of the 27 genome sequences were obtained from the NCBI ftp site (ftp://ftp.ncbi.nih.gov/genomes/) to generate a master genome table for gene annotation and COG analysis of the pan-genome and proteomic data sets. For functional pan-genomic comparison, the proteomic data sets of *M. vanbaalenii* PYR-1 (DSM 7251) treated with phthalate, the LMW PAHs fluorene, acenaphthylene, anthracene, and phenanthrene, and the HMW PAHs pyrene and benzo[a]pyrene, were used to select the genes which are expressed as proteins from the pan-genomic data [6-9,67,68].

### Genome comparison

Basic comparative genome analyses, including genome clustering based on COG information, were conducted using the JGI Integrated Microbial Genomes (IMG) system (https://img.jgi.doe.gov/cgi-bin/w/main.cgi). To visualize the function-based genomic comparison, the 27 mycobacterial genomes were clustered based on functional profiles; the PCA clustering analysis was conducted using the genome clustering function of the JGI IMG system (https://img.jgi.doe.gov/cgi-bin/w/main.cgi) with the condition: clustering type, COG and clustering method, Principal Components Analysis (PCA). Proximity of grouping indicates the similarity of genomes.

Pan-genome (core and pan-genome) and Venn diagram analyses of the pan-genomic data sets were conducted using EDGAR [78]. The core and pan-genome were computed by iterative pairwise comparison of a set of genomes. Using the metacontig function of EDGAR, we also defined custom groups of genomes for which the core genome or the pan genome have been stored as virtual contigs. A total of 9 virtual contigs according to the phenotype grouping of the 27 mycobacterial genomes was used in all subsequent EDGAR calculations, including Venn diagram analysis.

### Phylogenetic analysis

For a concatenated phylogenetic tree, we selected the 22 common genes from the core genome in the 27 mycobacterial strains [79] and estimated phylogenetic relationships. The 22 genes include (locus tags from *M.vanbaalenii* PYR-1); Mvan_0002, Mvan_1234, Mvan_1235, Mvan_1236, Mvan_1258, Mvan_1280, Mvan_1282, Mvan_1305, Mvan_1308, Mvan_1311, Mvan_1312, Mvan_1334, Mvan_1337, Mvan_1339, Mvan_1347, Mvan_1434, Mvan_1435, Mvan_1436, Mvan_1470, Mvan_1489, Mvan_2209, and Mvan_2651. Phylogenetic trees were constructed by the maximum likelihood method using PhyML [80] with the following parameters: (1) input data: the concatenated DNA sequences of the 22 common genes; (2) substitution model: HKY85 with substitution rate categories of 4 and estimated gamma shape parameter; (3) tree searching: BIONJ for starting tree and NINI for tree improvement with optimizing of topology and branch lengths; and (4) bootstrap: 100. MEGA4 [81] was used to visualize and manipulate the output trees.

### Gain/loss analysis of the PAH-degrading genes

The gain and loss of the PAH-degradation gene groups (a total of 23 groups) was assessed using the Count software [72] based on the PAH-degrading genes matrix across the genus Mycobacterium (Additional file 4: Table S13 and Additional file 5: Table S14). The Wagner parsimony model implemented in Count with the gain penalty of 1 was used to reconstruct gene content evolution in the history of the PAH-degrading gene groups as well as four types of the RHO group. The phylogenetic tree constructed by the maximum likelihood method (Figure 2A) was used as the guide topology.

### Ring hydroxylating oxygenase (RHO) classification

For classification and comparison of mycobacterial RHO enzymes, we used ClassRHO [67-69], which performs hierarchical cluster analysis using pairwise distance (PD) matrices and calculated from ClustalW default protein weight matrix (Blosum). For the classification of 246 RHO systems from the 27 mycobacterial strains, we used the automatic optimal threshold value that maximizes the learning accuracy. In the analysis, together with 42 standard RHO sequences, the amino acid sequences of large (α) subunits from the 246 RHO systems were used as RHO query sequences.

### Genomic island (GI) analysis

For computational identification, comparison, and visualization of genomic islands from the mycobacterial genomes, we used IslandViewer [82], a computational tool which integrates three different genomic island prediction methods, IslandPick [82], SIGI-HMM [83], and IslandPath-DIMOB [84].

### Network model of the relationships of the mycobacterial phenotypes

To model relationships and strengths among the nine phenotypes (Free-Living, PAH-degrading, Fast-growing, Non-pathogenic, Facultatively-host-associated, PAH-non-degrading, Pathogenic, Slow-growing, and Obligately intracellular), we reconstructed a network, Mycobacterial Phenotype Network (MPN). Basically, the network consists of nodes (vertices) and connections (edges) to represent mycobacteria and their relations, respectively. Since the phenotypes seem to be disjoint or individual sets, whose nodes (i.e. mycobacteria) can be partitioned into subsets, the structure between two groups (phenotypes) is loosely based on a bipartite graph. Seemingly, in the MPN several bipartite graphs are interrelated; however, different phenotypes may have identical mycobacteria, which are represented by individual nodes in each phenotype, to model the relationships. To represent the relationships, we connect the nodes between two phenotypes only if they are identical mycobacteria, i.e. the same mycobacterial occurrence/event between two phenotypes. In the MPN, the connection is undirected since it includes no distinction or causality between the nodes. Intuitively, in the MPN, the greater the number of edges between groups, the stronger the connection is. We define the connection strength between two groups, Gx and Gy, as CS (Gx, Gy), defined as

$$ CS\left(Gx,\; Gy\right)=\left[\uprho (Gx)+\uprho (Gy)\right]*50 $$

where ρ (Gx) is the percentage of nodes in Gx with edges to Gy.

For visualizing and analyzing networks in this study, Cytoscape [77] was used. In the MPN, the thickness of edge between two phenotype groups is based on the degree of the connection strength (CS value).

### Availability of supporting data

The data sets supporting the phylogenetic result of this article are available on the TreeBASE repository, ID: S16971, http://purl.org/phylo/treebase/phylows/study/TB2:S16971.

## Additional files

Additional file 1: Table S1. Core-genome analysis of the six PAH-degrading mycobacteria. **Table S2**. Functional analysis of the 3,533-gene core-genome. **Table S3**. Analysis of 136 genes involved in PAH metabolism. **Table S4**. Protein expression profile of 136 genes in *M. vanbaalenii* PYR-1. **Table S5**. List of 113 genes out of the 136 genes, which are expressed as proteins involved in PAH degradation pathways. Additional file 2: Table S6. Analysis of HGT in the six PAH-degrading mycobacteria. **Table S7**. List of 754 proteins contributing to the core-genome from a total of 3,533 genes. **Table S8**. List of 2,124 proteins contributing to the dispensable genome from a total of 5,999 genes. **Table S9**. List of 29 proteins, based on COG categories, responsible for all enzymatic reactions involved in the degradation of PAHs in *M. vanbaalenii* PYR-1. **Table S10**. List of 29 proteins, based on functional modules, responsible for all enzymatic reactions involved in the degradation of PAHs in *M. vanbaalenii* PYR-1. Additional file 3: Table S11. List of 248 core genes involved in PAH degradation identified in *M. vanbaalenii* PYR-1. **Table S12**. Summary of 248 core genes. Additional file 4: Table S13. Distribution of PAH-degrading genes across the genus *Mycobacterium*. Additional file 5: Table S14. Distribution of RHO enzymes across the genus *Mycobacterium*.

## Abbreviations

PAHs: Polycyclic aromatic hydrocarbons
HMW: High-molecular-weight
LMW: Low-molecular-weight
HGT: Horizontal gene transfer
PAH-MN: PAH Metabolic network
MPN: Mycobacterial phenotype network
FL: Free-living
FHA: Facultatively-host-associated
OI: Obligately intracellular
RHO: Ring-hydroxylating oxygenase
ETC: Electron transfer component
PN: Phenotype network
COG: Cluster of orthologous groups
CYP: Cytochrome P450 monooxygenase
RCD: Ring-cleavage dioxygenase
PCA: Principal components analysis
PD: Pairwise distance
NCBI: National Center for Biotechnology Information
IMG: Integrated Microbial Genomes
JGI: Joint Genome Institute
